# Commentary: Evaluation of the real-world safety of eptifibatide in the treatment of ARDS: results of a disproportionality analysis of FAERS data

**DOI:** 10.3389/fmed.2026.1835916

**Published:** 2026-04-29

**Authors:** Ying Feng, Liu-Cheng Li, Xiao-Fang Niu, Hai-Hong Lin

**Affiliations:** 1 Department of Pharmacy, Heze Municipal Hospital, Heze, China; 2 Department of Pharmacy, Sir Run Run Shaw Hospital, Zhejiang University School of Medicine, Hangzhou, China; 3 Department of Pharmacy, Wenling TCM Hospital Affiliated to Zhejiang Chinese Medical University, Wenling, China

**Keywords:** antiplatelet therapy, bleeding risk management, dynamic systems approach, pharmacist-led intervention, pharmacovigilance

Antiplatelet therapy (such as eptifibatide) forces a precarious balance, where maximizing protection from arterial thrombosis inevitably increases the hazard of life-threatening bleeding. We read with great interest the article by Zhang et al. evaluating the real-world safety of eptifibatide using FDA Adverse Event Reporting System data, which provides valuable insights into both known and previously unreported adverse events (1). While the authors employed rigorous disproportionality analyses to generate safety signals, we would like to highlight the critical blind spots inherent to such pharmacovigilance studies. We agree on the need for vigilance but propose evolving from a static view of bleeding to a dynamic systems approach. We here propose perspectives to address blind spots and chart future research.

## The blind spot of causal inference and unexplored exposome of bleeding risk

The absence of detailed clinical context such as concomitant medications, underlying comorbidities, or temporal relationship nuances, restrains the ability to disentangle drug-induced effects from confounding by indication or disease progression. This is particularly relevant in critically ill populations with acute respiratory distress syndrome (ARDS), where the interplay between the disease itself, multi-organ dysfunction, and polypharmacy creates a complex web of potential etiologies for bleeding and thrombotic events. The databases capture a snapshot of an event but not the subsequent cascade of clinical decisions and outcomes. This represents a critical blind spot due to the lack of connection in the temporal event chain. Future research must employ advanced causal inference methods using longitudinal real-world data from hospitals to model these trajectories. The critical question is “what is the net clinical benefit when accounting for the post-bleed adjustment of therapy and long-term ischemic outcomes.” This requires analyzing the entire patient journey. While eptifibatide's antiplatelet mechanism is well-characterized, the actual hemorrhagic risk in clinical practice is likely modulated by a constellation of exposures, including concomitant anticoagulants, renal function, genetic polymorphisms affecting platelet receptor responsiveness, and even procedural variables such as vascular access site management. Current pharmacovigilance analyses overlook cumulative drug exposure and unrecorded factors like over-the-counter medications, herbal supplements, renal trajectory, and social determinants of health (e.g., geographic access to care) (2–5), all of which profoundly modify bleeding risk.

## Translating the dynamic systems approach into practice: a hypothetical case

To move beyond conceptual advocacy, we illustrate our proposed framework with a concrete clinical scenario. Consider a 70-year-old patient with acute coronary syndrome and underlying chronic kidney disease (eGFR 35 mL/min) who is prescribed eptifibatide alongside dual antiplatelet therapy (aspirin and clopidogrel). The patient also self-reports taking a Ginkgo biloba supplement, an interaction often invisible in spontaneous reporting systems like FAERS. Under the static signal approach, a pharmacist reviewing the FAERS database would note a disproportionate bleeding signal for eptifibatide but would lack the granular data to assess this patient's individual risk. The response would be a general warning: “monitor for bleeding.” In contrast, a dynamic systems approach would integrate multiple data streams: (1) real-time renal function trajectory from the electronic health record (EHR); (2) pharmacogenomic data (e.g., CYP2C19 loss-of-function status affecting clopidogrel metabolism); (3) cumulative drug exposure accounting for the herbal supplement; and (4) temporal patterns of platelet aggregation tests. A machine learning model trained on longitudinal hospital data could generate a personalized bleeding risk trajectory, alerting the clinical pharmacist when the risk exceeds a dynamic threshold. The pharmacist's intervention would then follow a structured pathway: (a) causal assessment by distinguishing drug-drug interaction (eptifibatide-ginkgo) from renal-mediated toxicity; (b) shared decision-making by presenting visualized trade-off curves to the patient (e.g., “stopping eptifibatide reduces bleeding risk by X% but increases 30-day stent thrombosis risk by Y%”); (c) implementation by adjusting the infusion protocol and substituting the herbal supplement; and (d) longitudinal follow-up by monitoring for post-bleed ischemic events using RE-AIM framework indicators. This workflow transforms the pharmacist from a passive signal receiver into an active system integrator, addressing precisely the “blind spot of disconnected temporal event chains” highlighted in our manuscript. This hypothetical case explicitly maps each limitation identified in our critique to a corresponding solution: (1) the absence of temporal data in FAERS is addressed by longitudinal EHR-based causal inference; (2) the lack of granular clinical context is overcome by integrating pharmacogenomic and renal function data; (3) unrecorded exposures (e.g., herbal supplements) are captured through dynamic medication reconciliation; and (4) disconnected event chains are remedied by structured pharmacist workflows guided by implementation frameworks like RE-AIM (Reach, Effectiveness, Adoption, Implementation, Maintenance) (6).

## Unraveling the complexity of pharmacist interventions

As we know, pharmacists act as guardians of medication safety, clinical contributors, patient educators, interdisciplinary collaborators, and innovators advancing personalized pharmacotherapy (7–12). According to the authors' findings, pharmacists accounted for 27.16% of adverse event reports, ranking second only to physicians (42.04%). This substantial contribution highlights the increasingly critical role that pharmacists play in post-marketing pharmacovigilance. Unlike physicians, pharmacists are often positioned at the intersection of medication dispensing, administration monitoring, and patient education, making them uniquely sensitive to medication errors, dosing issues, and early signs of adverse drug reactions. However, the current study does not delve into the qualitative complexity of pharmacist-reported events. Pharmacist-generated reports may capture distinct safety signals compared to those from other healthcare professionals, such as medication preparation errors, infusion-related complications, or drug-drug interactions that manifest in real-time clinical settings. Yet, the FAERS database does not allow for granular exploration of the clinical contexts, interventions, or reasoning behind pharmacist submissions. To ensure real-world effectiveness, future studies should use implementation frameworks like RE-AIM to evaluate intervention effectiveness across diverse populations, contexts, and long-term system costs (6). We advocate for a stepped-wedge cluster randomized trial across tertiary, community, and rural hospitals, testing a pharmacist-led antithrombosis stewardship program with primary outcomes of guideline-concordant management within 6 h and 30-day major adverse cardiovascular event rates, alongside a cost-effectiveness analysis. This transforms the pharmacist's role from passive signal recipient to active, data-driven decision architect.

## A blueprint for the next decade of research

To move from critique to construction, we explicitly link each blind spot identified above to a corresponding research priority in the roadmap below. The future risk prediction lies in fusing real-world data with pharmacogenomic data. To improve dynamic risk prediction, future models should apply gradient-boosted decision tree algorithms (e.g., XGBoost, LightGBM) to longitudinal electronic health record data. By integrating time-varying covariates including daily serum creatinine (capturing acute kidney injury trajectories), hemoglobin drop rates, and sequential organ failure assessment scores, these models could estimate the conditional probability of post-bleeding ischemic events (e.g., stent thrombosis, recurrent myocardial infarction) over a 72-h window. This approach would allow clinicians to target interventions to patients at highest dynamic risk, shifting pharmacovigilance from static signal detection to a real-time, actionable decision support system. To operationalize pharmacogenomics in bleeding management, we propose prioritizing clinically actionable variants with known effect sizes. Specifically, CYP2C19 loss-of-function alleles (^*^2, ^*^3), which impair clopidogrel activation and increase thrombotic risk, along with ABCB1 polymorphisms that modulate intestinal absorption of ticagrelor and rivaroxaban, should be integrated into a point-of-care genotyping workflow. Pharmacists could then compute a combined pharmacogenetic risk score to guide decisions on platelet transfusion thresholds or the selection of reversal agents (e.g., andexanet alfa vs. vitamin K), thereby moving beyond mechanistic exploration toward a clear, actionable algorithm for genotype-guided bleeding reversal.

Besides, the vision is not for pharmacists to work in parallel, but for their expertise to be woven into the clinical workflow. This involves developing real-time automated bleeding risk alerts linked to standardized intervention pathways (e.g., automatic pharmacist consultation for high-risk patients on interacting medications). Furthermore, the decision to continue or stop antiplatelet therapy is a preference-sensitive one. To explore how pharmacists can facilitate shared decision-making, future research should develop and test risk communication tools that help patients visualize thrombotic vs. bleeding trade-offs while incorporating their values. This moves the pharmacist's role from pure drug management to humanistic management, addressing the psychological impact of even minor bleeds on a patient's quality of life, treatment confidence, and long-term resilience. Fourth, the complexity of managing these patients across different specialties (neurology for stroke, cardiology for acute coronary syndrome, respiratory for pulmonary embolism) calls for an integrated model. We advocate for the establishment of multidisciplinary, pharmacist-led antithrombosis clinics. These clinics, staffed by specialized pharmacists, could provide unified, protocol-driven care, manage perioperative bridging, and conduct long-term follow-up, thereby ensuring continuity and closing the loop between hospital and community.

In conclusion, FAERS-based pharmacovigilance provides essential static safety signals, but its inherent limitations like lack of temporal causality, missing clinical and exposome context, static risk prediction, and fragmented intervention pathways, require a paradigm shift. We have explicitly mapped each of these blind spots to a corresponding solution, e.g. causal inference with longitudinal data, machine learning with time-varying covariates, pharmacogenomic integration, and pharmacist-led multidisciplinary clinics. By forging these explicit connections, we can empower pharmacists to move beyond simply detecting bleeding signals and toward actively managing dynamic, patient-centered treatment resilience.
